# Supplementary material for: The triglycerides-glucose index and the triglycerides to high-density lipoprotein cholesterol ratio are both effective predictors of in-hospital death in non-diabetic patients with AMI
Source: PeerJ. 2022 Nov 21;10:e14346. doi: 10.7717/peerj.14346 (PMC9686411; doi:10.7717/peerj.14346)

Normality test of death and no-death groups in STEMI patients

Usually there are two test methods for normal distribution, one is Shapiro-Wilk test, which is suitable for small sample data (sample size ≤ 5000); another is Kolmogorov–Smirnov test, which is suitable for large sample data (sample size). >5000). If it is significant (p<0.05 or 0.01), it means that the null hypothesis is rejected (the data conforms to the normal distribution), the data does not satisfy the normal distribution, otherwise, the data conforms to the normal distribution. It is usually difficult to meet the test in real research situations. If the absolute value of the sample kurtosis is less than 10 and the absolute value of the skewness is less than 3, the data can be described as basically conforming to the normal distribution combined with QQ map.

(1) age

| **Tests of Normality** | | | | | | | |
| --- | --- | --- | --- | --- | --- | --- | --- |
|  | Death | Kolmogorov-Smirnov^a^ | | | Shapiro-Wilk | | |
|  |  | Statistic | df | Sig. | Statistic | df | Sig. |
| Age | No | .070 | 829 | .000 | .988 | 829 | .000 |
|  | Yes | .159 | 45 | .006 | .911 | 45 | .002 |
| a. Lilliefors Significance Correction | | | | | | | |

|  | Death | Kurtosis | Skewness |
| --- | --- | --- | --- |
| Age | No | -0.715 | -0.087 |
|  | Yes | 0.606 | -1.031 |


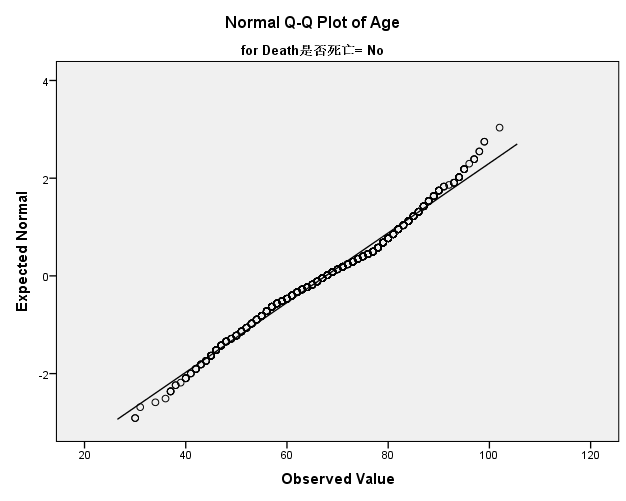

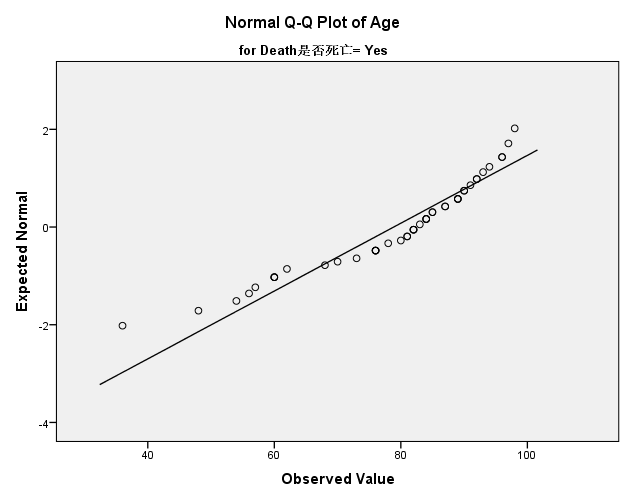


(2) SBP

| **Tests of Normality** | | | | | | | |
| --- | --- | --- | --- | --- | --- | --- | --- |
|  | Death | Kolmogorov-Smirnov^a^ | | | Shapiro-Wilk | | |
|  |  | Statistic | df | Sig. | Statistic | df | Sig. |
| SBP | No | .065 | 825 | .000 | .984 | 825 | .000 |
|  | Yes | .094 | 44 | .200^*^ | .955 | 44 | .083 |
| *. This is a lower bound of the true significance. | | | | | | | |
| a. Lilliefors Significance Correction | | | | | | | |

|  | Death | Kurtosis | Skewness |
| --- | --- | --- | --- |
| SBP | No | 1.361 | 0.093 |
|  | Yes | 1.324 | 0.867 |


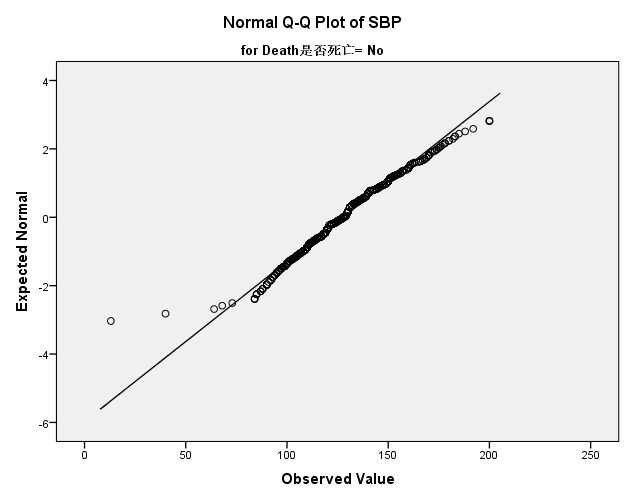

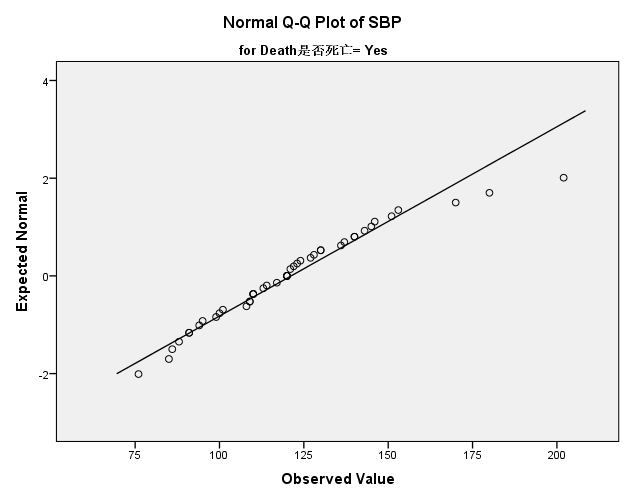


(3) Neutrophil

| **Tests of Normality** | | | | | | | |
| --- | --- | --- | --- | --- | --- | --- | --- |
|  | Death | Kolmogorov-Smirnov^a^ | | | Shapiro-Wilk | | |
|  |  | Statistic | df | Sig. | Statistic | df | Sig. |
| Neut | No | .095 | 787 | .000 | .873 | 787 | .000 |
|  | Yes | .105 | 43 | .200^*^ | .941 | 43 | .028 |
| *. This is a lower bound of the true significance. | | | | | | | |
| a. Lilliefors Significance Correction | | | | | | | |

|  | Death | Kurtosis | Skewness |
| --- | --- | --- | --- |
| Neutrophil | No | 9.644 | 2.008 |
|  | Yes | -0.042 | 0.754 |


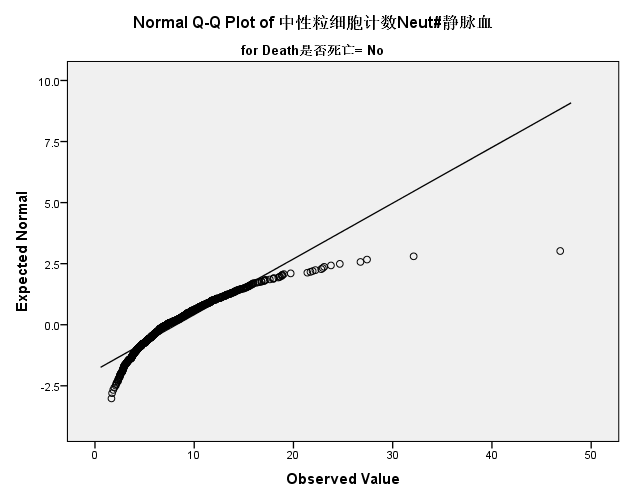

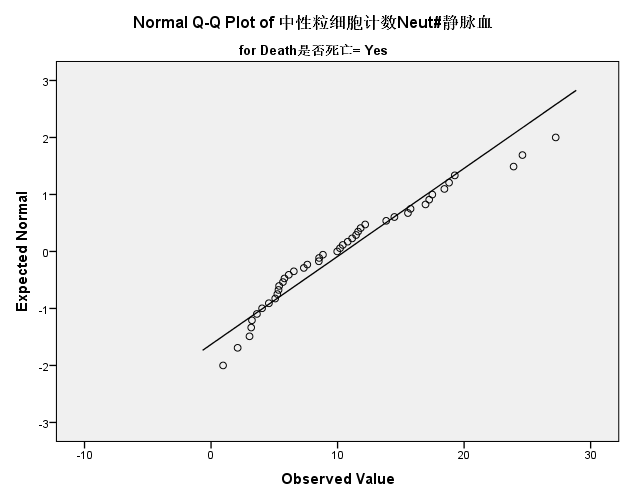


(4) Lymphocyte

| **Tests of Normality** | | | | | | | |
| --- | --- | --- | --- | --- | --- | --- | --- |
|  | Death | Kolmogorov-Smirnov^a^ | | | Shapiro-Wilk | | |
|  |  | Statistic | df | Sig. | Statistic | df | Sig. |
| Lymph | No | .087 | 787 | .000 | .927 | 787 | .000 |
|  | Yes | .184 | 43 | .001 | .840 | 43 | .000 |
| a. Lilliefors Significance Correction | | | | | | | |

|  | Death | Kurtosis | Skewness |
| --- | --- | --- | --- |
| Lymphocyte | No | 2.676 | 1.242 |
|  | Yes | 3.760 | 1.761 |


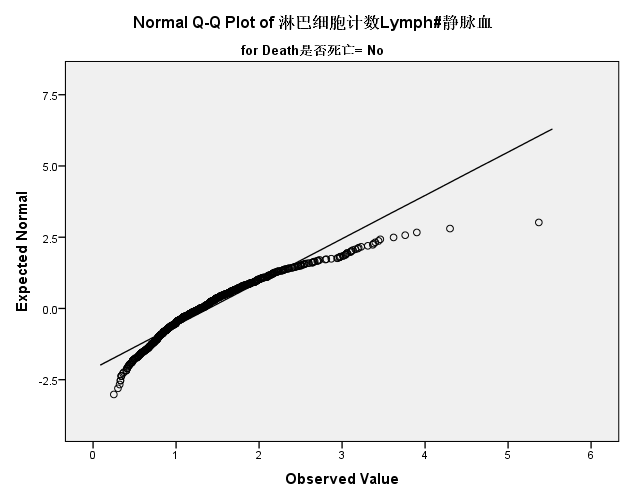

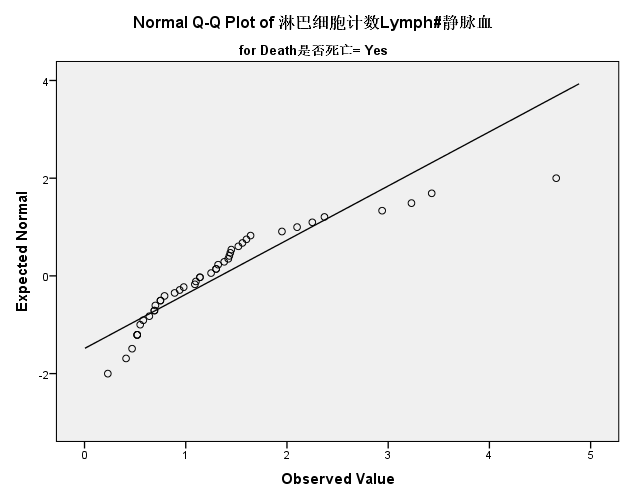


(5) WBC

| **Tests of Normality** | | | | | | | |
| --- | --- | --- | --- | --- | --- | --- | --- |
|  | Death | Kolmogorov-Smirnov^a^ | | | Shapiro-Wilk | | |
|  |  | Statistic | df | Sig. | Statistic | df | Sig. |
| WBC | No | .087 | 787 | .000 | .873 | 787 | .000 |
|  | Yes | .108 | 43 | .200^*^ | .942 | 43 | .032 |
| *. This is a lower bound of the true significance. | | | | | | | |
| a. Lilliefors Significance Correction | | | | | | | |

|  | Death | Kurtosis | Skewness |
| --- | --- | --- | --- |
| WBC | No | 10.299 | 2.071 |
|  | Yes | 0.030 | 0.760 |


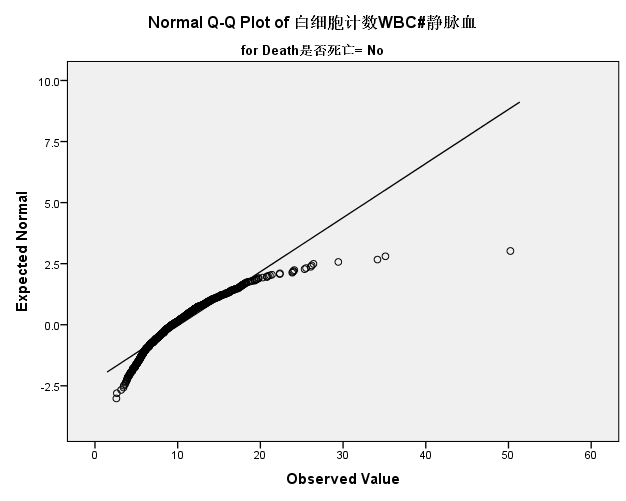

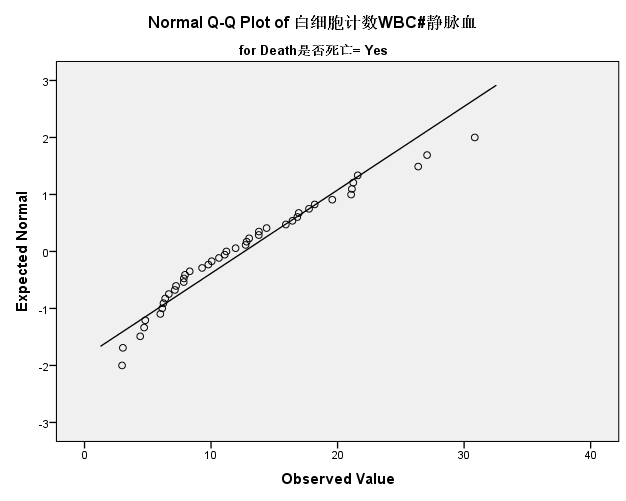


(6) PLT

| **Tests of Normality** | | | | | | | |
| --- | --- | --- | --- | --- | --- | --- | --- |
|  | Death | Kolmogorov-Smirnov^a^ | | | Shapiro-Wilk | | |
|  |  | Statistic | df | Sig. | Statistic | df | Sig. |
| PLT | No | .050 | 787 | .000 | .966 | 787 | .000 |
|  | Yes | .124 | 43 | .093 | .971 | 43 | .346 |
| a. Lilliefors Significance Correction | | | | | | | |

|  | Death | Kurtosis | Skewness |
| --- | --- | --- | --- |
| PLT | No | 3.246 | 0.864 |
|  | Yes | 0.069 | 0.444 |


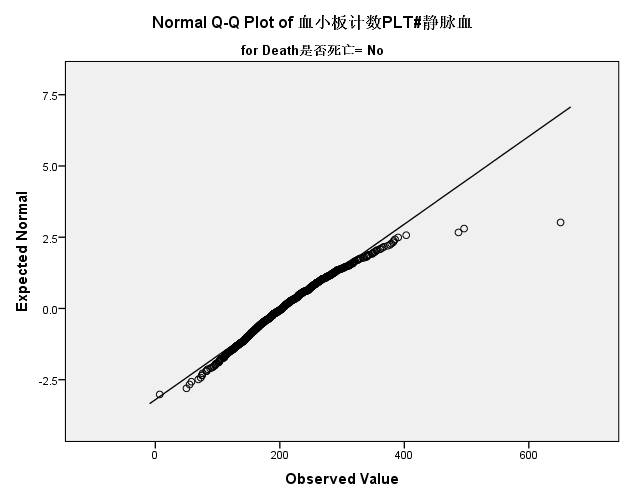

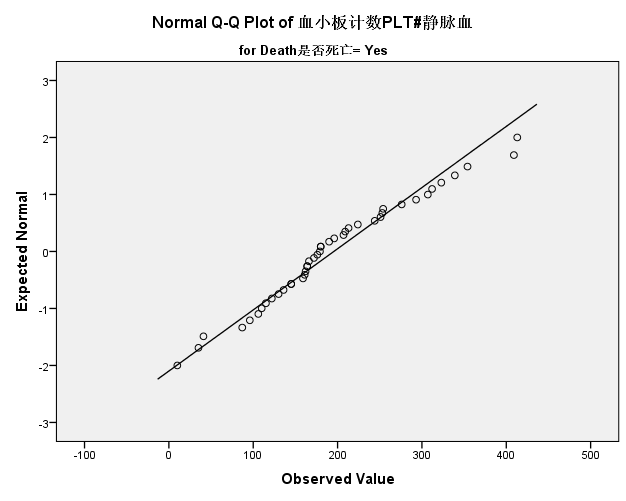


(7) Hb

| **Tests of Normality** | | | | | | | |
| --- | --- | --- | --- | --- | --- | --- | --- |
|  | Death | Kolmogorov-Smirnov^a^ | | | Shapiro-Wilk | | |
|  |  | Statistic | df | Sig. | Statistic | df | Sig. |
| Hb | No | .052 | 787 | .000 | .983 | 787 | .000 |
|  | Yes | .074 | 43 | .200^*^ | .986 | 43 | .859 |
| *. This is a lower bound of the true significance. | | | | | | | |
| a. Lilliefors Significance Correction | | | | | | | |

|  | Death | Kurtosis | Skewness |
| --- | --- | --- | --- |
| Hb | No | 0.927 | -0.542 |
|  | Yes | -0.334 | -0.085 |


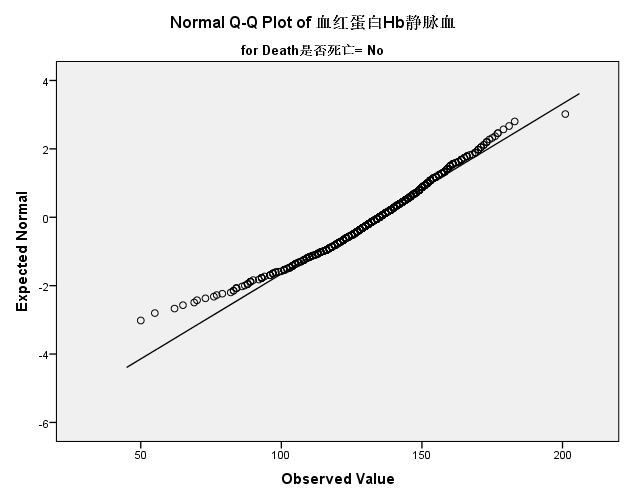

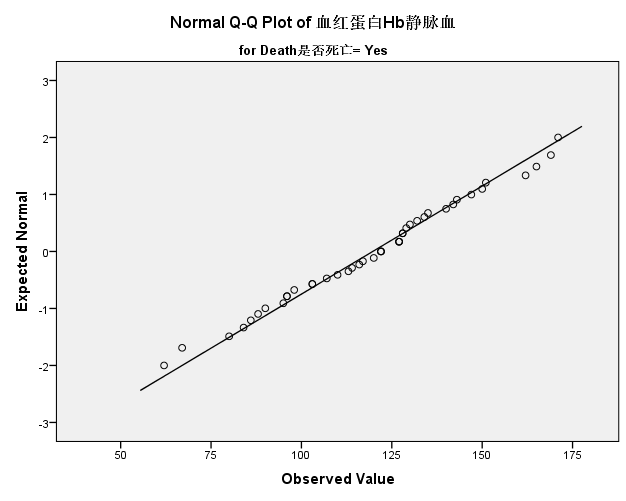


(8) ALT

| **Tests of Normality** | | | | | | | |
| --- | --- | --- | --- | --- | --- | --- | --- |
|  | Death | Kolmogorov-Smirnov^a^ | | | Shapiro-Wilk | | |
|  |  | Statistic | df | Sig. | Statistic | df | Sig. |
| ALT | No | .282 | 825 | .000 | .301 | 825 | .000 |
|  | Yes | .343 | 45 | .000 | .422 | 45 | .000 |
| a. Lilliefors Significance Correction | | | | | | | |

|  | Death | Kurtosis | Skewness |
| --- | --- | --- | --- |
| ALT | No | 301.975 | 14.915 |
|  | Yes | 18.571 | 4.162 |


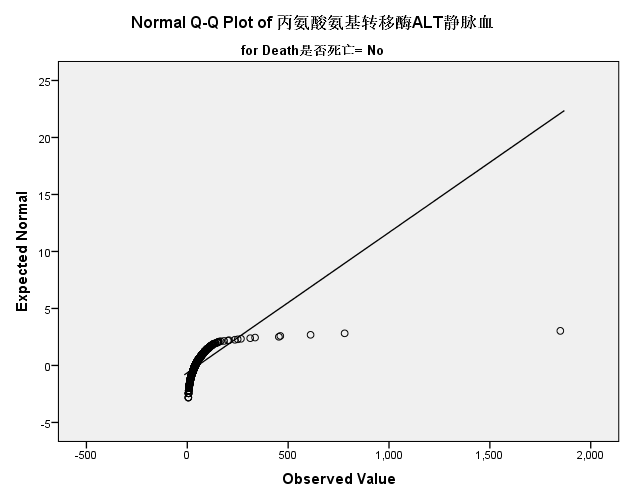

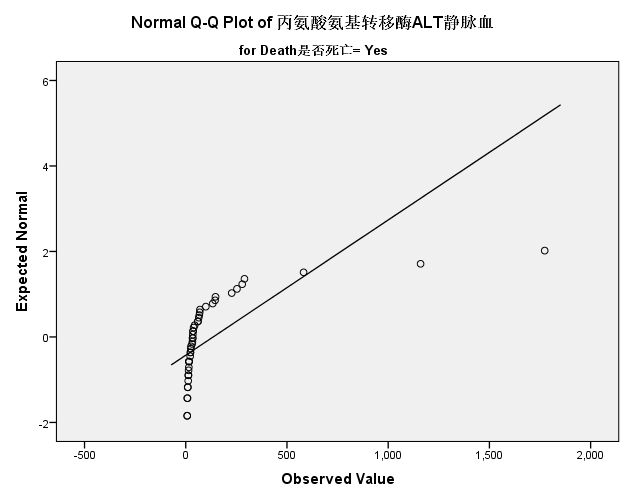


(9) AST

| **Tests of Normality** | | | | | | | |
| --- | --- | --- | --- | --- | --- | --- | --- |
|  | Death | Kolmogorov-Smirnov^a^ | | | Shapiro-Wilk | | |
|  |  | Statistic | df | Sig. | Statistic | df | Sig. |
| AST | No | .176 | 825 | .000 | .803 | 825 | .000 |
|  | Yes | .288 | 45 | .000 | .559 | 45 | .000 |
| a. Lilliefors Significance Correction | | | | | | | |

|  | Death | Kurtosis | Skewness |
| --- | --- | --- | --- |
| AST | No | 5.558 | 1.966 |
|  | Yes | 17.915 | 3.761 |


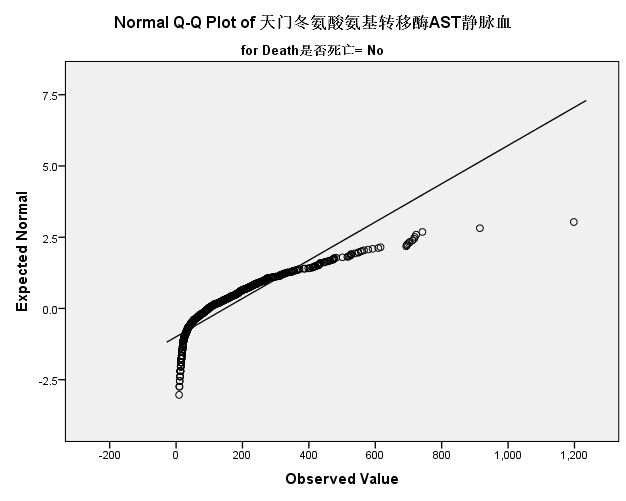

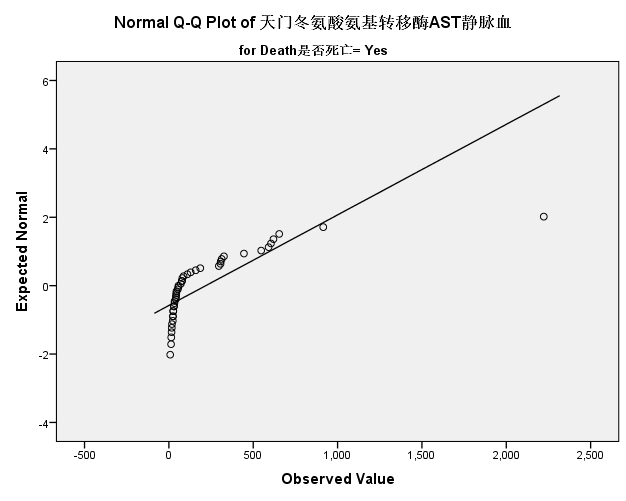


(10) LDH

| **Tests of Normality** | | | | | | | |
| --- | --- | --- | --- | --- | --- | --- | --- |
|  | Death | Kolmogorov-Smirnov^a^ | | | Shapiro-Wilk | | |
|  |  | Statistic | df | Sig. | Statistic | df | Sig. |
| LDH | No | .152 | 824 | .000 | .799 | 824 | .000 |
|  | Yes | .286 | 45 | .000 | .519 | 45 | .000 |
| a. Lilliefors Significance Correction | | | | | | | |

|  | Death | Kurtosis | Skewness |
| --- | --- | --- | --- |
| LDH | No | 11.551 | 2.399 |
|  | Yes | 21.560 | 4.252 |


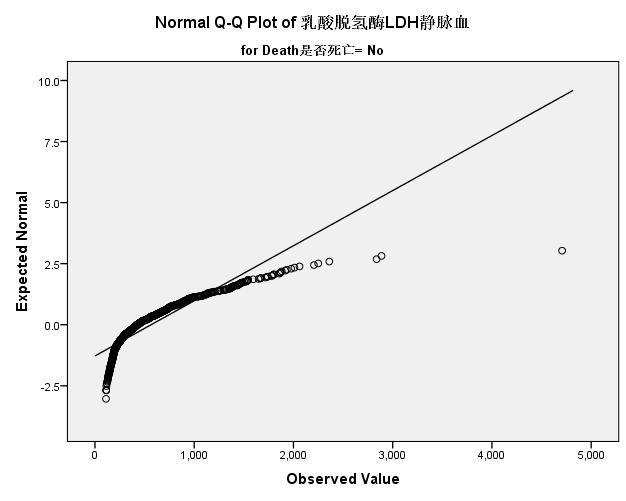

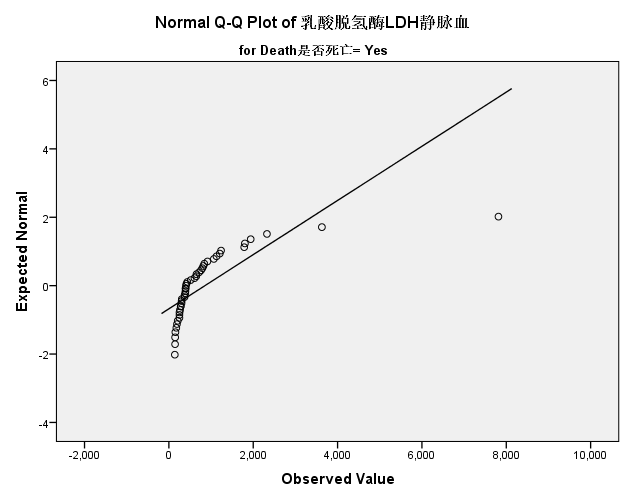


(11) LDL-C

| **Tests of Normality** | | | | | | | |
| --- | --- | --- | --- | --- | --- | --- | --- |
|  | Death | Kolmogorov-Smirnov^a^ | | | Shapiro-Wilk | | |
|  |  | Statistic | df | Sig. | Statistic | df | Sig. |
| LDLC | No | .056 | 806 | .000 | .968 | 806 | .000 |
|  | Yes | .118 | 41 | .167 | .976 | 41 | .532 |
| a. Lilliefors Significance Correction | | | | | | | |

|  | Death | Kurtosis | Skewness |
| --- | --- | --- | --- |
| LDL-C | No | 2.454 | 0.807 |
|  | Yes | -0.368 | 0.040 |


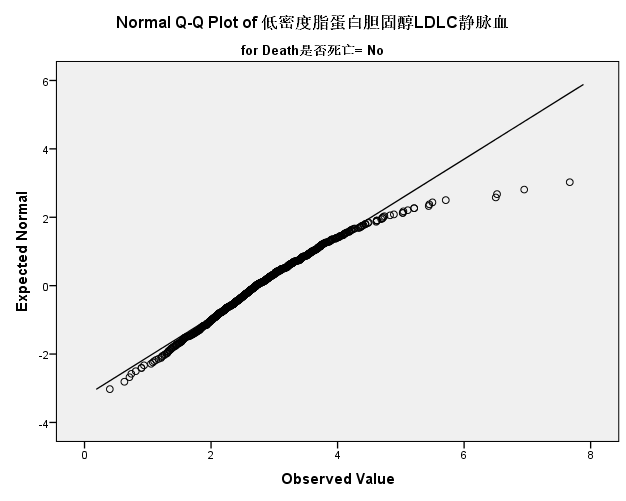

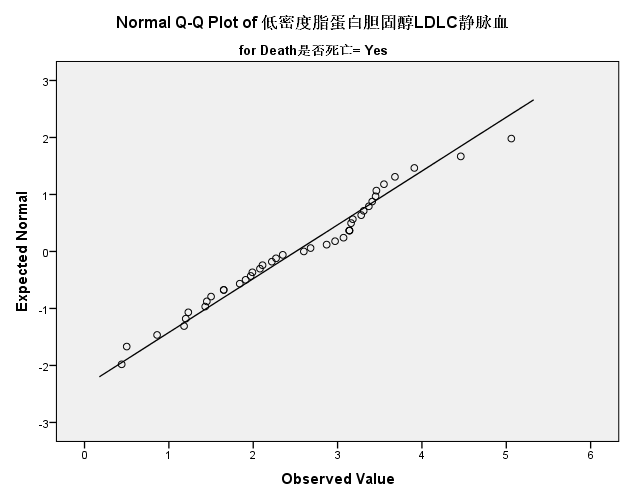


(12) HDL-C

| **Tests of Normality** | | | | | | | |
| --- | --- | --- | --- | --- | --- | --- | --- |
|  | Death | Kolmogorov-Smirnov^a^ | | | Shapiro-Wilk | | |
|  |  | Statistic | df | Sig. | Statistic | df | Sig. |
| HDLC | No | .070 | 806 | .000 | .960 | 806 | .000 |
|  | Yes | .088 | 41 | .200^*^ | .979 | 41 | .630 |
| *. This is a lower bound of the true significance. | | | | | | | |
| a. Lilliefors Significance Correction | | | | | | | |

|  | Death | Kurtosis | Skewness |
| --- | --- | --- | --- |
| HDL-C | No | 1.682 | 0.896 |
|  | Yes | -0.310 | -0.310 |


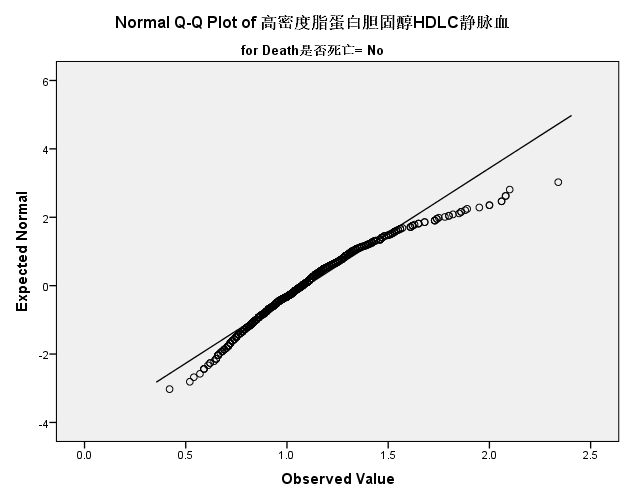

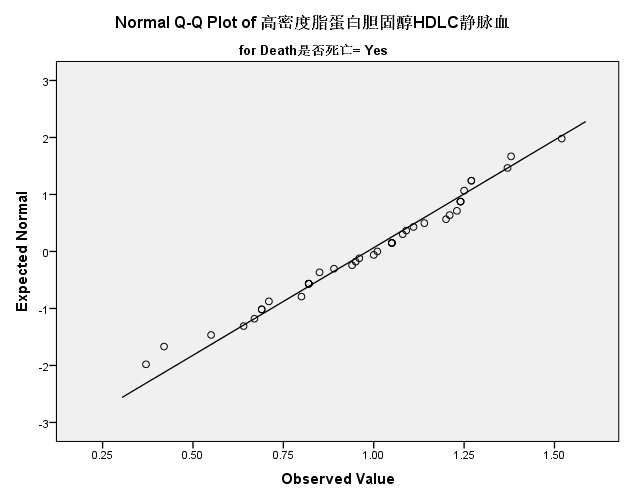


(13) BUN

| **Tests of Normality** | | | | | | | |
| --- | --- | --- | --- | --- | --- | --- | --- |
|  | Death | Kolmogorov-Smirnov^a^ | | | Shapiro-Wilk | | |
|  |  | Statistic | df | Sig. | Statistic | df | Sig. |
| BUN | No | .200 | 828 | .000 | .631 | 828 | .000 |
|  | Yes | .241 | 45 | .000 | .803 | 45 | .000 |
| a. Lilliefors Significance Correction | | | | | | | |

|  | Death | Kurtosis | Skewness |
| --- | --- | --- | --- |
| BUN | No | 33.906 | 33.906 |
|  | Yes | 1.295 | 1.438 |


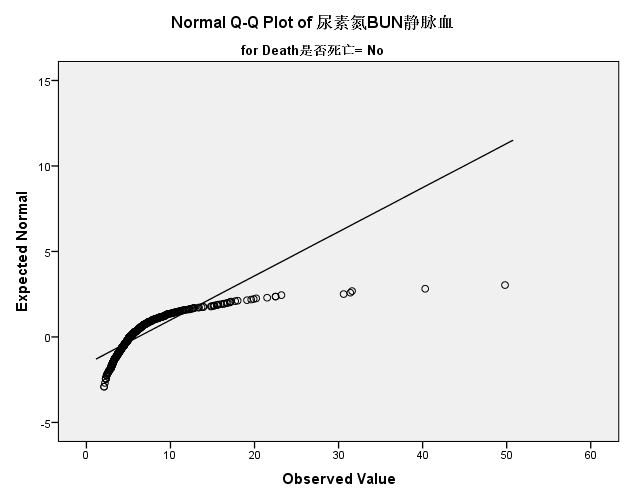

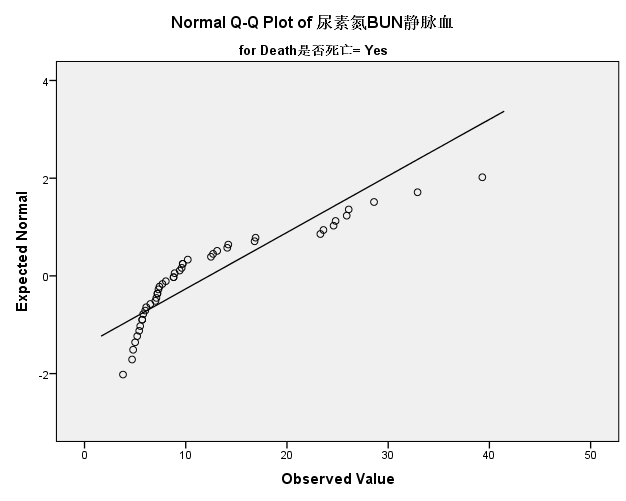


(14) UA

| **Tests of Normality** | | | | | | | |
| --- | --- | --- | --- | --- | --- | --- | --- |
|  | Death | Kolmogorov-Smirnov^a^ | | | Shapiro-Wilk | | |
|  |  | Statistic | df | Sig. | Statistic | df | Sig. |
| UA | No | .075 | 829 | .000 | .944 | 829 | .000 |
|  | Yes | .144 | 45 | .021 | .892 | 45 | .001 |
| a. Lilliefors Significance Correction | | | | | | | |

|  | Death | Kurtosis | Skewness |
| --- | --- | --- | --- |
| UA | No | 3.50 | 1.139 |
|  | Yes | 4.994 | 1.577 |


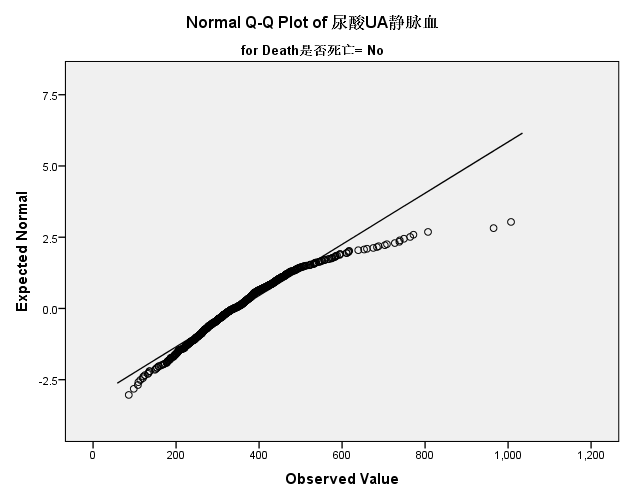


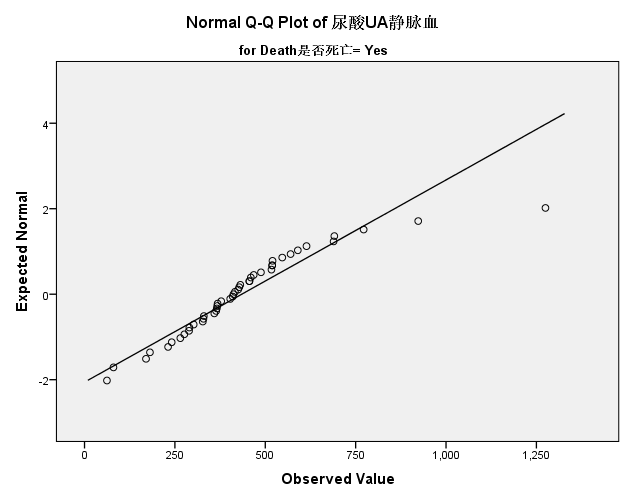


(15) cTNI

| **Tests of Normality** | | | | | | | |
| --- | --- | --- | --- | --- | --- | --- | --- |
|  | Death | Kolmogorov-Smirnov^a^ | | | Shapiro-Wilk | | |
|  |  | Statistic | df | Sig. | Statistic | df | Sig. |
| cTnI | No | .297 | 544 | .000 | .494 | 544 | .000 |
|  | Yes | .303 | 34 | .000 | .544 | 34 | .000 |
| a. Lilliefors Significance Correction | | | | | | | |

|  | Death | Kurtosis | Skewness |
| --- | --- | --- | --- |
| cTNI | No | 57.956 | 6.265 |
|  | Yes | 10.260 | 3.163 |


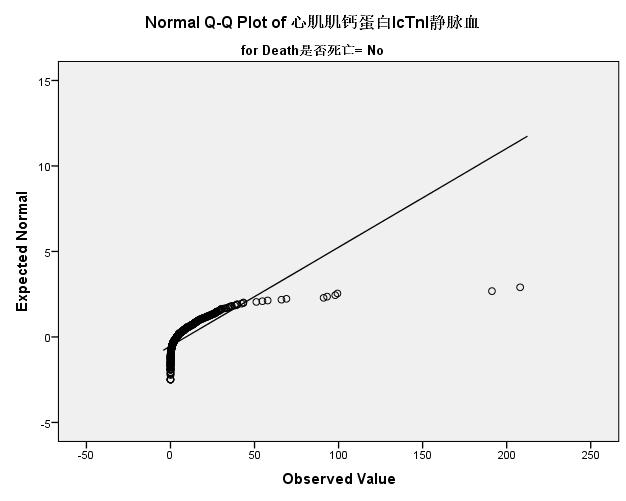

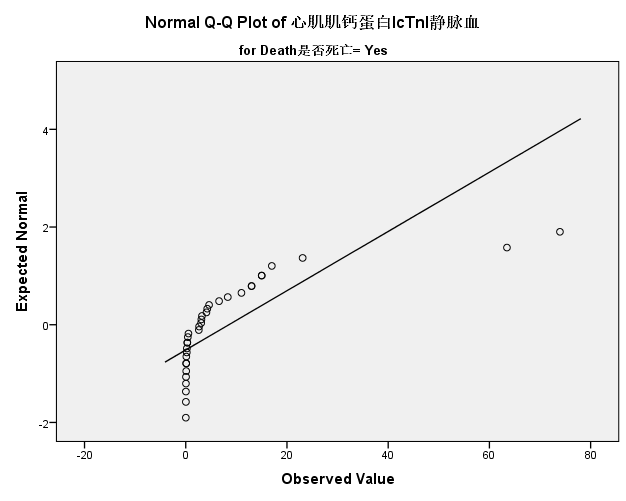


(16) TC

| **Tests of Normality** | | | | | | | |
| --- | --- | --- | --- | --- | --- | --- | --- |
|  | Death | Kolmogorov-Smirnov^a^ | | | Shapiro-Wilk | | |
|  |  | Statistic | df | Sig. | Statistic | df | Sig. |
| TC | No | .049 | 829 | .000 | .975 | 829 | .000 |
|  | Yes | .071 | 45 | .200^*^ | .969 | 45 | .256 |
| *. This is a lower bound of the true significance. | | | | | | | |
| a. Lilliefors Significance Correction | | | | | | | |

|  | Death | Kurtosis | Skewness |
| --- | --- | --- | --- |
| TC | No | 1.197 | 0.694 |
|  | Yes | 0.243 | 0.476 |


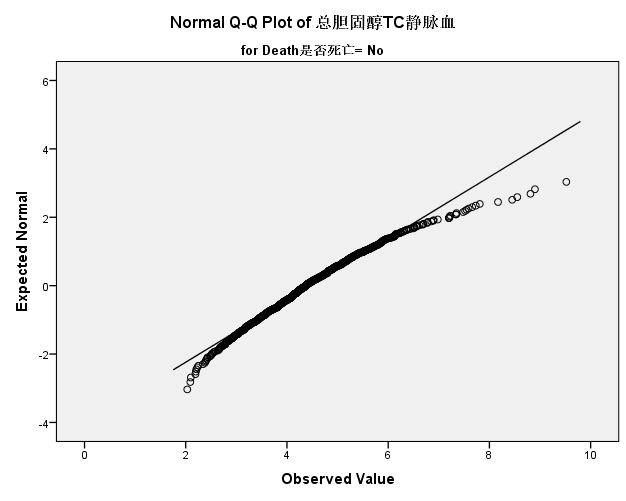

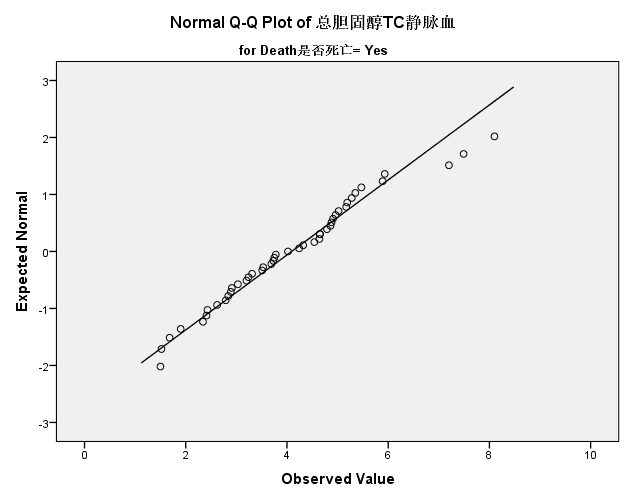


(17) ALB

| **Tests of Normality** | | | | | | | |
| --- | --- | --- | --- | --- | --- | --- | --- |
|  | Death | Kolmogorov-Smirnov^a^ | | | Shapiro-Wilk | | |
|  |  | Statistic | df | Sig. | Statistic | df | Sig. |
| ALB | No | .050 | 825 | .000 | .984 | 825 | .000 |
|  | Yes | .125 | 45 | .075 | .969 | 45 | .258 |
| a. Lilliefors Significance Correction | | | | | | | |

|  | Death | Kurtosis | Skewness |
| --- | --- | --- | --- |
| ALB | No | 0.861 | -0.530 |
|  | Yes | 1.045 | -0.106 |


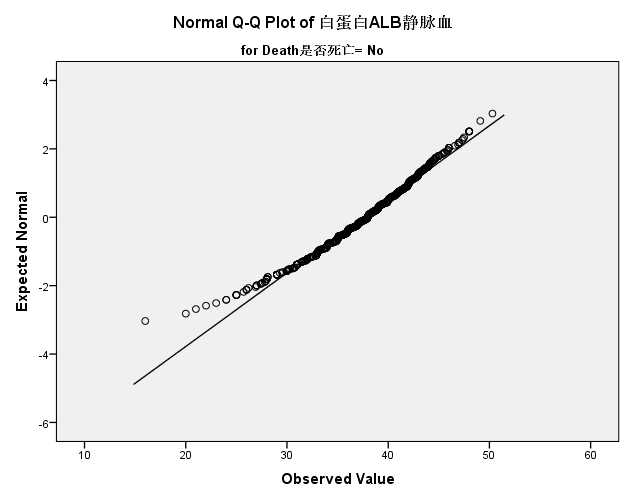

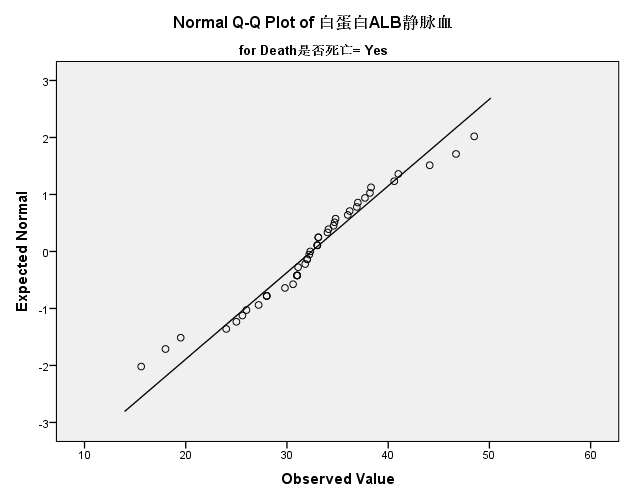


(18) creatinine

| **Tests of Normality** | | | | | | | |
| --- | --- | --- | --- | --- | --- | --- | --- |
|  | Death | Kolmogorov-Smirnov^a^ | | | Shapiro-Wilk | | |
|  |  | Statistic | df | Sig. | Statistic | df | Sig. |
| Creatinine | No | .268 | 829 | .000 | .405 | 829 | .000 |
|  | Yes | .227 | 45 | .000 | .599 | 45 | .000 |
| a. Lilliefors Significance Correction | | | | | | | |

|  | Death | Kurtosis | Skewness |
| --- | --- | --- | --- |
| Creatinine | No | 67.959 | 7.340 |
|  | Yes | 20.254 | 3.956 |


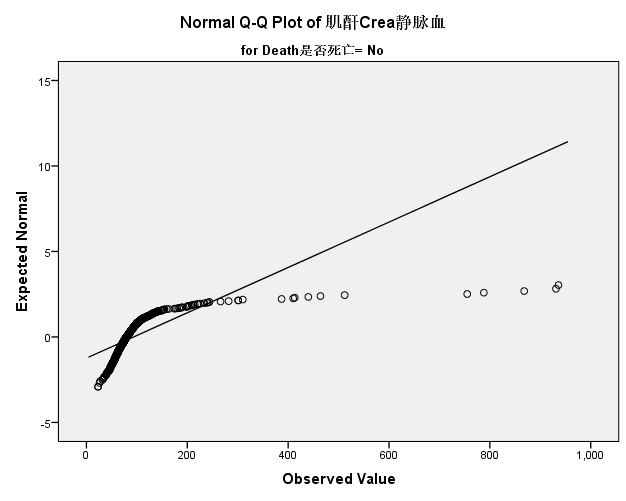

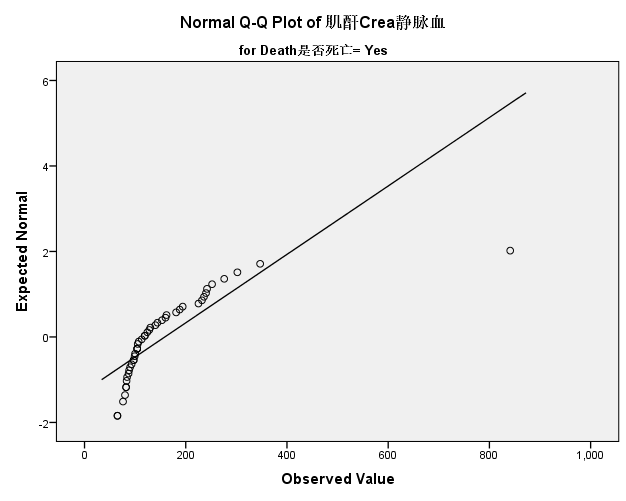


(19) GLU

| **Tests of Normality** | | | | | | | |
| --- | --- | --- | --- | --- | --- | --- | --- |
|  | Death | Kolmogorov-Smirnov^a^ | | | Shapiro-Wilk | | |
|  |  | Statistic | df | Sig. | Statistic | df | Sig. |
| Glu | No | .123 | 829 | .000 | .827 | 829 | .000 |
|  | Yes | .264 | 45 | .000 | .521 | 45 | .000 |
| a. Lilliefors Significance Correction | | | | | | | |

|  | Death | Kurtosis | Skewness |
| --- | --- | --- | --- |
| GLU | No | 8.981 | 2.292 |
|  | Yes | 25.273 | 4.575 |


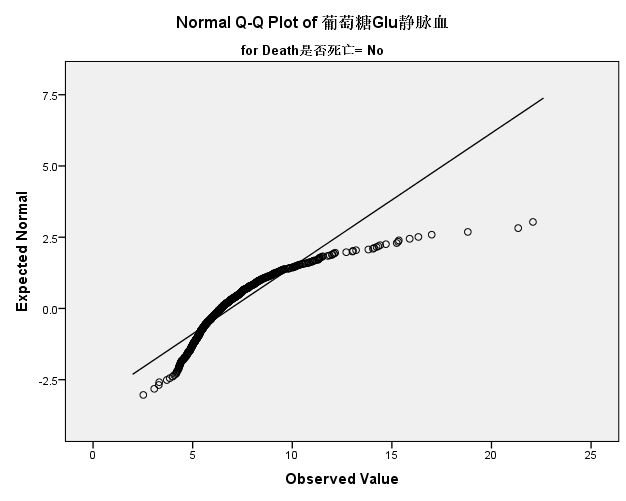

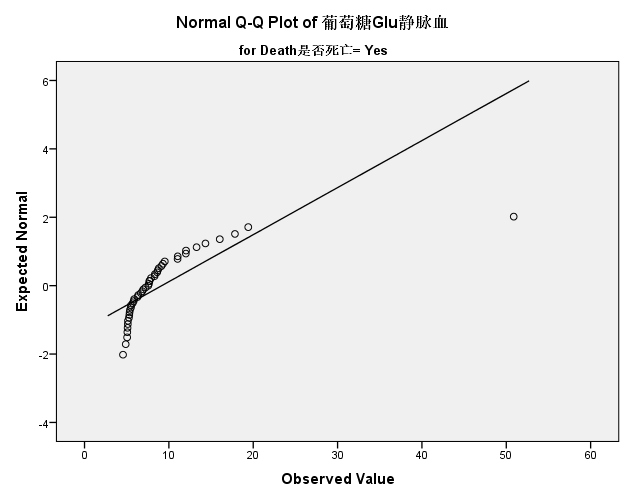


(20) hsCRP

| **Tests of Normality** | | | | | | | |
| --- | --- | --- | --- | --- | --- | --- | --- |
|  | Death | Kolmogorov-Smirnov^a^ | | | Shapiro-Wilk | | |
|  |  | Statistic | df | Sig. | Statistic | df | Sig. |
| hsCRP | No | .240 | 216 | .000 | .704 | 216 | .000 |
|  | Yes | .216 | 27 | .002 | .835 | 27 | .001 |
| a. Lilliefors Significance Correction | | | | | | | |

|  | Death | Kurtosis | Skewness |
| --- | --- | --- | --- |
| hsCRP | No | 4.668 | 2.177 |
|  | Yes | 0.633 | 1.218 |


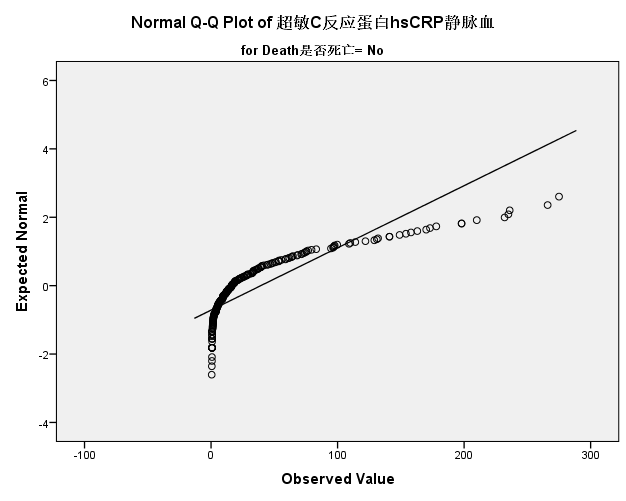

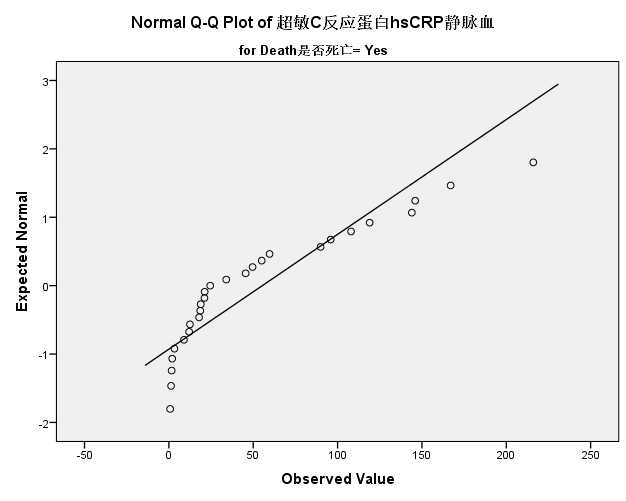


(21) HbA1C

| **Tests of Normality** | | | | | | | |
| --- | --- | --- | --- | --- | --- | --- | --- |
|  | Death | Kolmogorov-Smirnov^a^ | | | Shapiro-Wilk | | |
|  |  | Statistic | df | Sig. | Statistic | df | Sig. |
| HbA1c | No | .162 | 143 | .000 | .826 | 143 | .000 |
|  | Yes | .274 | 5 | .200^*^ | .842 | 5 | .171 |
| *. This is a lower bound of the true significance. | | | | | | | |
| a. Lilliefors Significance Correction | | | | | | | |

|  | Death | Kurtosis | Skewness |
| --- | --- | --- | --- |
| HbA1C | No | 5.772 | 2.047 |
|  | Yes | 2.885 | 1.607 |


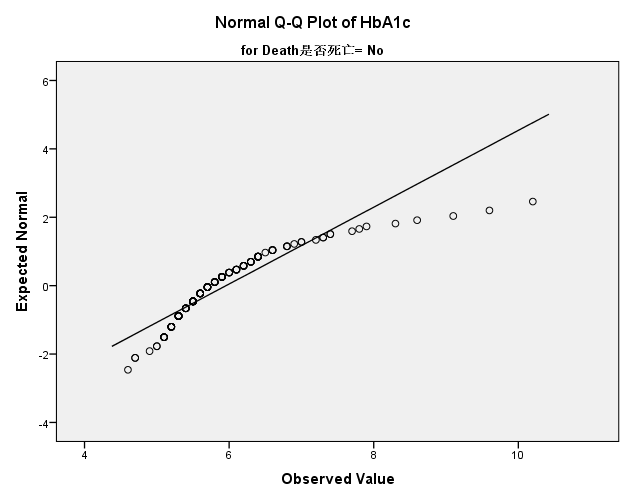

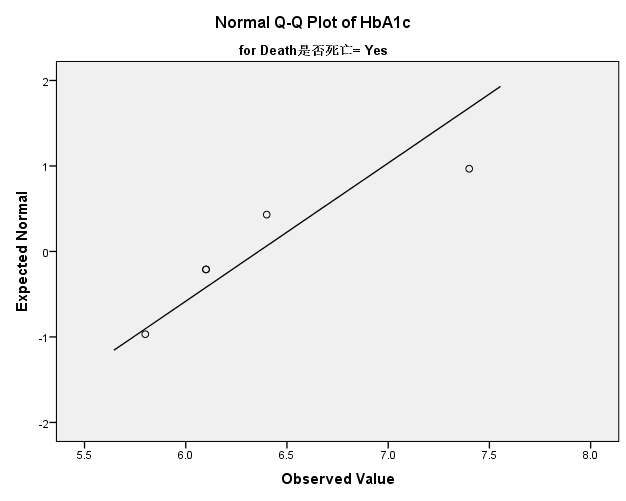


(22) LVEF

| **Tests of Normality** | | | | | | | |
| --- | --- | --- | --- | --- | --- | --- | --- |
|  | Death | Kolmogorov-Smirnov^a^ | | | Shapiro-Wilk | | |
|  |  | Statistic | df | Sig. | Statistic | df | Sig. |
| EF | No | .052 | 663 | .000 | .991 | 663 | .001 |
|  | Yes | .093 | 20 | .200^*^ | .968 | 20 | .710 |
| *. This is a lower bound of the true significance. | | | | | | | |
| a. Lilliefors Significance Correction | | | | | | | |

|  | Death | Kurtosis | Skewness |
| --- | --- | --- | --- |
| LVEF | No | -0.348 | -0.348 |
|  | Yes | -0.674 | -0.276 |


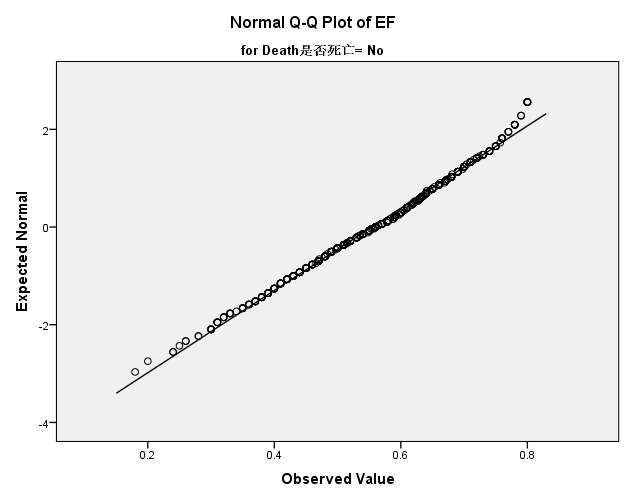

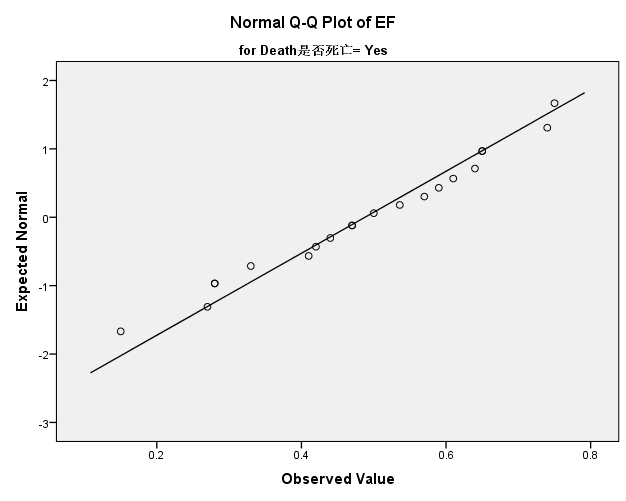


(23) TyG

| **Tests of Normality** | | | | | | | |
| --- | --- | --- | --- | --- | --- | --- | --- |
|  | Death | Kolmogorov-Smirnov^a^ | | | Shapiro-Wilk | | |
|  |  | Statistic | df | Sig. | Statistic | df | Sig. |
| TyG | No | .031 | 829 | .051 | .994 | 829 | .001 |
|  | Yes | .199 | 45 | .000 | .868 | 45 | .000 |
| a. Lilliefors Significance Correction | | | | | | | |

|  | Death | Kurtosis | Skewness |
| --- | --- | --- | --- |
| TyG | No | 0.499 | 0.141 |
|  | Yes | 0.484 | 1.148 |


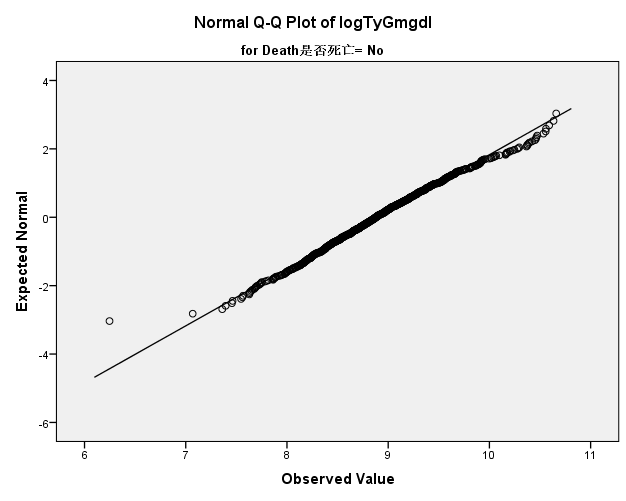

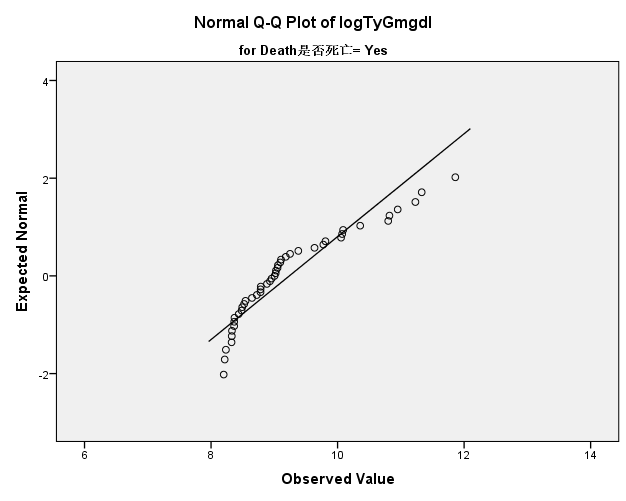


(24) TG/HDL-C

| **Tests of Normality** | | | | | | | |
| --- | --- | --- | --- | --- | --- | --- | --- |
|  | Death | Kolmogorov-Smirnov^a^ | | | Shapiro-Wilk | | |
|  |  | Statistic | df | Sig. | Statistic | df | Sig. |
| TGHDL | No | .133 | 806 | .000 | .814 | 806 | .000 |
|  | Yes | .212 | 41 | .000 | .581 | 41 | .000 |
| a. Lilliefors Significance Correction | | | | | | | |

|  | Death | Kurtosis | Skewness |
| --- | --- | --- | --- |
| TyG | No | 14.987 | 2.651 |
|  | Yes | 20.937 | 4.110 |


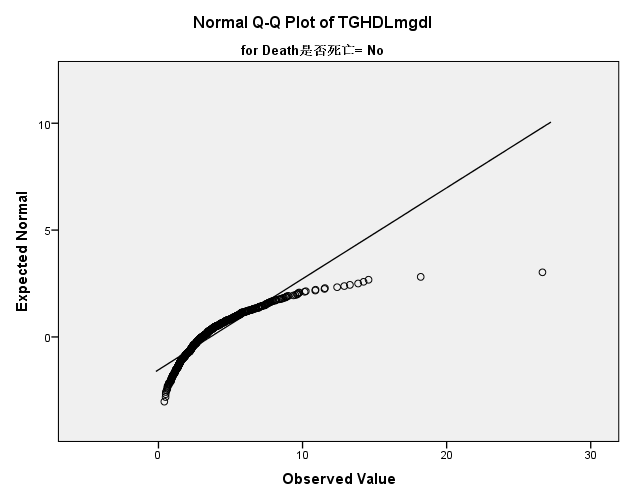

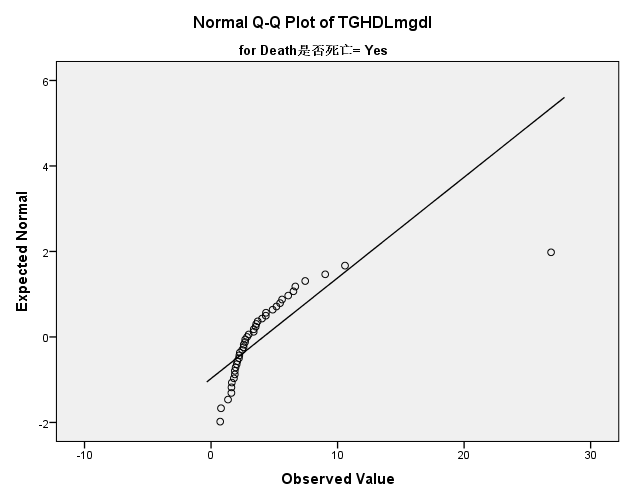

Supplement: Supplemental Information 3 [file peerj-10-14346-s003.docx]
