# Supplementary material for: The triglycerides-glucose index and the triglycerides to high-density lipoprotein cholesterol ratio are both effective predictors of in-hospital death in non-diabetic patients with AMI
Source: PeerJ. 2022 Nov 21;10:e14346. doi: 10.7717/peerj.14346 (PMC9686411; doi:10.7717/peerj.14346)
Supplement: Supplemental Information 5 [file peerj-10-14346-s005.docx]

1. Homogeneity test for variance of NSTEMI

| **Independent Samples Test** | | | | | | | | | | |
| --- | --- | --- | --- | --- | --- | --- | --- | --- | --- | --- |
|  | | Levene's Test for Equality of Variances | | t-test for Equality of Means | | | | | | |
|  |  | F | Sig. | t | df | Sig. (2-tailed) | Mean Difference | Std. Error Difference | 95% Confidence Interval of the Difference | |
|  |  |  |  |  |  |  |  |  | Lower | Upper |
| Age | Equal variances assumed | 29.481 | .000 | -5.955 | 772 | .000 | -12.525 | 2.103 | -16.655 | -8.396 |
|  | Equal variances not assumed |  |  | -11.178 | 59.191 | .000 | -12.525 | 1.121 | -14.767 | -10.283 |
| SBP | Equal variances assumed | 2.817 | .094 | 3.248 | 765 | .001 | 11.999 | 3.694 | 4.747 | 19.251 |
|  | Equal variances not assumed |  |  | 2.678 | 40.690 | .011 | 11.999 | 4.481 | 2.947 | 21.051 |
| Neut | Equal variances assumed | 13.271 | .000 | -3.966 | 726 | .000 | -2.32648 | .58658 | -3.47807 | -1.17489 |
|  | Equal variances not assumed |  |  | -2.567 | 40.697 | .014 | -2.32648 | .90635 | -4.15731 | -.49565 |
| Lymph | Equal variances assumed | .865 | .353 | 2.733 | 726 | .006 | .30925 | .11317 | .08708 | .53142 |
|  | Equal variances not assumed |  |  | 2.599 | 43.152 | .013 | .30925 | .11897 | .06935 | .54915 |
| PLT | Equal variances assumed | 2.654 | .104 | 1.412 | 727 | .158 | 16.222 | 11.488 | -6.331 | 38.774 |
|  | Equal variances not assumed |  |  | 1.243 | 42.471 | .221 | 16.222 | 13.048 | -10.103 | 42.546 |
| Hb | Equal variances assumed | 1.341 | .247 | 5.609 | 727 | .000 | 20.583 | 3.669 | 13.379 | 27.786 |
|  | Equal variances not assumed |  |  | 4.817 | 42.278 | .000 | 20.583 | 4.273 | 11.961 | 29.205 |
| LDLC | Equal variances assumed | .076 | .783 | 2.639 | 750 | .008 | .39842 | .15099 | .10201 | .69484 |
|  | Equal variances not assumed |  |  | 2.663 | 41.123 | .011 | .39842 | .14960 | .09634 | .70051 |
| HDLC | Equal variances assumed | 5.200 | .023 | 2.082 | 750 | .038 | .09345 | .04489 | .00532 | .18157 |
|  | Equal variances not assumed |  |  | 1.652 | 39.388 | .106 | .09345 | .05655 | -.02090 | .20780 |
| UA | Equal variances assumed | 6.021 | .014 | -1.146 | 772 | .252 | -23.013 | 20.074 | -62.420 | 16.394 |
|  | Equal variances not assumed |  |  | -.907 | 41.555 | .370 | -23.013 | 25.374 | -74.236 | 28.211 |
| TC | Equal variances assumed | .665 | .415 | 2.390 | 772 | .017 | 17.39475 | 7.27724 | 3.10922 | 31.68029 |
|  | Equal variances not assumed |  |  | 2.266 | 42.859 | .029 | 17.39475 | 7.67748 | 1.91017 | 32.87934 |
| ALB | Equal variances assumed | 9.102 | .003 | 5.874 | 771 | .000 | 4.8223 | .8209 | 3.2108 | 6.4338 |
|  | Equal variances not assumed |  |  | 4.523 | 41.405 | .000 | 4.8223 | 1.0661 | 2.6699 | 6.9747 |
| EF | Equal variances assumed | 3.664 | .056 | 3.391 | 583 | .001 | .09597820 | .02830403 | .04038792 | .15156848 |
|  | Equal variances not assumed |  |  | 2.662 | 21.983 | .014 | .09597820 | .03604931 | .02121315 | .17074325 |

1. Homogeneity test for variance of STEMI

| **Independent Samples Test** | | | | | | | | | | |
| --- | --- | --- | --- | --- | --- | --- | --- | --- | --- | --- |
|  | | Levene's Test for Equality of Variances | | t-test for Equality of Means | | | | | | |
|  |  | F | Sig. | t | df | Sig. (2-tailed) | Mean Difference | Std. Error Difference | 95% Confidence Interval of the Difference | |
|  |  |  |  |  |  |  |  |  | Lower | Upper |
| Age | Equal variances assumed | .138 | .710 | -5.220 | 872 | .000 | -11.214 | 2.148 | -15.430 | -6.998 |
|  | Equal variances not assumed |  |  | -5.093 | 48.631 | .000 | -11.214 | 2.202 | -15.639 | -6.789 |
| SBP | Equal variances assumed | 1.790 | .181 | 1.927 | 867 | .054 | 6.443 | 3.344 | -.120 | 13.006 |
|  | Equal variances not assumed |  |  | 1.627 | 46.200 | .111 | 6.443 | 3.961 | -1.528 | 14.414 |
| Neut | Equal variances assumed | 17.637 | .000 | -3.347 | 828 | .001 | -2.36371 | .70623 | -3.74992 | -.97750 |
|  | Equal variances not assumed |  |  | -2.366 | 44.127 | .022 | -2.36371 | .99923 | -4.37737 | -.35005 |
| Lymph | Equal variances assumed | 3.673 | .056 | .497 | 828 | .620 | .05226 | .10523 | -.15429 | .25881 |
|  | Equal variances not assumed |  |  | .375 | 44.472 | .710 | .05226 | .13951 | -.22882 | .33334 |
| PLT | Equal variances assumed | 11.578 | .001 | 1.174 | 828 | .241 | 12.257 | 10.441 | -8.238 | 32.752 |
|  | Equal variances not assumed |  |  | .852 | 44.259 | .399 | 12.257 | 14.394 | -16.748 | 41.261 |
| Hb | Equal variances assumed | 7.035 | .008 | 4.238 | 828 | .000 | 13.588 | 3.206 | 7.294 | 19.881 |
|  | Equal variances not assumed |  |  | 3.327 | 44.711 | .002 | 13.588 | 4.084 | 5.361 | 21.814 |
| LDLC | Equal variances assumed | 6.830 | .009 | 2.128 | 845 | .034 | .29774 | .13994 | .02307 | .57240 |
|  | Equal variances not assumed |  |  | 1.770 | 42.750 | .084 | .29774 | .16820 | -.04153 | .63701 |
| HDLC | Equal variances assumed | .378 | .539 | 2.708 | 845 | .007 | .11407 | .04212 | .03140 | .19675 |
|  | Equal variances not assumed |  |  | 2.690 | 44.104 | .010 | .11407 | .04241 | .02862 | .19953 |
| UA | Equal variances assumed | 24.996 | .000 | -4.702 | 872 | .000 | -85.180 | 18.117 | -120.738 | -49.623 |
|  | Equal variances not assumed |  |  | -2.683 | 45.333 | .010 | -85.180 | 31.744 | -149.102 | -21.258 |
| TC | Equal variances assumed | 11.330 | .001 | 2.236 | 872 | .026 | 14.97941 | 6.69984 | 1.82972 | 28.12911 |
|  | Equal variances not assumed |  |  | 1.686 | 46.571 | .099 | 14.97941 | 8.88660 | -2.90248 | 32.86130 |
| ALB | Equal variances assumed | 5.187 | .023 | 7.031 | 868 | .000 | 5.1270 | .7292 | 3.6958 | 6.5581 |
|  | Equal variances not assumed |  |  | 5.158 | 46.425 | .000 | 5.1270 | .9940 | 3.1267 | 7.1273 |
| EF | Equal variances assumed | 6.088 | .014 | 2.428 | 681 | .015 | .06633575 | .02731817 | .01269778 | .11997371 |
|  | Equal variances not assumed |  |  | 1.764 | 19.585 | .093 | .06633575 | .03759912 | -.01220134 | .14487283 |
