# Supplementary material for: The triglycerides-glucose index and the triglycerides to high-density lipoprotein cholesterol ratio are both effective predictors of in-hospital death in non-diabetic patients with AMI
Source: PeerJ. 2022 Nov 21;10:e14346. doi: 10.7717/peerj.14346 (PMC9686411; doi:10.7717/peerj.14346)
Supplement: Supplemental Information 6 [file peerj-10-14346-s006.docx]

Supplemental material 1 Collinearity analysis of variables included in the variate logistic regression of TyG.

| **Coefficients^a^** | | | | | | | | |
| --- | --- | --- | --- | --- | --- | --- | --- | --- |
| Model | | Unstandardized Coefficients | | Standardized Coefficients | t | Sig. | Collinearity Statistics | |
|  |  | B | Std. Error | Beta |  |  | Tolerance | VIF |
| 1 | (Constant) | -.409 | .105 |  | -3.889 | .000 |  |  |
|  | Age | .002 | .000 | .121 | 4.189 | .000 | .660 | 1.514 |
|  | SBP | -.001 | .000 | -.077 | -3.125 | .002 | .908 | 1.101 |
|  | ALT | .000 | .000 | .083 | 3.398 | .001 | .936 | 1.068 |
|  | BUN | .011 | .002 | .228 | 5.930 | .000 | .373 | 2.684 |
|  | UA | -6.747E-5 | .000 | -.037 | -1.322 | .186 | .705 | 1.418 |
|  | ALB | -.004 | .001 | -.095 | -3.330 | .001 | .677 | 1.476 |
|  | Cr | .000 | .000 | -.047 | -1.429 | .153 | .501 | 1.995 |
|  | WBC | .004 | .001 | .074 | 2.982 | .003 | .906 | 1.104 |
|  | Hb | .000 | .000 | -.039 | -1.333 | .183 | .638 | 1.568 |
|  | TC | .000 | .000 | -.024 | -.929 | .353 | .844 | 1.185 |
|  | TyG | .065 | .009 | .180 | 7.144 | .000 | .866 | 1.154 |
| a. Dependent Variable: Death or No-death | | | | | | | | |

Supplemental material 2 Collinearity analysis of variables included in the variate logistic regression of TG/HDL-C.

| **Coefficients^a^** | | | | | | | | |
| --- | --- | --- | --- | --- | --- | --- | --- | --- |
| Model | | Unstandardized Coefficients | | Standardized Coefficients | t | Sig. | Collinearity Statistics | |
|  |  | B | Std. Error | Beta |  |  | Tolerance | VIF |
| 1 | (Constant) | .087 | .078 |  | 1.112 | .267 |  |  |
|  | Age | .002 | .000 | .116 | 3.768 | .000 | .618 | 1.619 |
|  | SBP | -.001 | .000 | -.072 | -2.847 | .004 | .913 | 1.096 |
|  | ALT | .000 | .000 | .083 | 3.337 | .001 | .943 | 1.060 |
|  | BUN | .011 | .002 | .239 | 5.964 | .000 | .365 | 2.738 |
|  | UA | -8.413E-5 | .000 | -.047 | -1.625 | .104 | .702 | 1.424 |
|  | ALB | -.004 | .001 | -.085 | -2.902 | .004 | .680 | 1.470 |
|  | Cr | .000 | .000 | -.045 | -1.313 | .190 | .498 | 2.009 |
|  | WBC | .004 | .001 | .082 | 3.229 | .001 | .909 | 1.100 |
|  | Hb | .000 | .000 | -.040 | -1.321 | .187 | .634 | 1.577 |
|  | TC | 3.601E-5 | .000 | .007 | .283 | .778 | .885 | 1.130 |
|  | TG/HDL-C | .009 | .002 | .097 | 3.734 | .000 | .865 | 1.156 |
| a. Dependent Variable: Death or No-death | | | | | | | | |
