# Supplementary material for: The triglycerides-glucose index and the triglycerides to high-density lipoprotein cholesterol ratio are both effective predictors of in-hospital death in non-diabetic patients with AMI
Source: PeerJ. 2022 Nov 21;10:e14346. doi: 10.7717/peerj.14346 (PMC9686411; doi:10.7717/peerj.14346)
Supplement: Supplemental Information 7 [file peerj-10-14346-s007.docx]

**Multivariate logistic regression of “Forward” and “Backward” methods**

Supplemental table 1 Variate logistic regression of TG/HDL-C by “Forward stepwise (LR)” method.

|  | B | S.E. | Wald | df | Sig. | Exp(B) | 95% CI for EXP(B) | |
| --- | --- | --- | --- | --- | --- | --- | --- | --- |
|  |  |  |  |  |  |  | Lower | Upper |
| Age (years) | .069 | .013 | 27.714 | 1 | .000 | 1.072 | 1.044 | 1.100 |
| ALT (U/L) | .001 | .000 | 4.197 | 1 | .040 | 1.001 | 1.000 | 1.002 |
| ALB (g/L) | -.080 | .026 | 9.580 | 1 | .002 | .923 | .878 | .971 |
| BUN (mmol/L) | .066 | .019 | 11.756 | 1 | .001 | 1.068 | 1.029 | 1.110 |
| SBP (mmHg) | -.017 | .006 | 8.130 | 1 | .004 | .983 | .972 | .995 |
| WBC (109/L) | .062 | .023 | 6.955 | 1 | .008 | 1.063 | 1.016 | 1.113 |
| TG/HDL-C | .149 | .047 | 10.029 | 1 | .002 | 1.161 | 1.059 | 1.273 |

Supplemental table 2 Variate logistic regression of TG/HDL-C by “Backward stepwise (LR)” method.

|  | B | S.E. | Wald | df | Sig. | Exp(B) | 95% CI for EXP(B) | |
| --- | --- | --- | --- | --- | --- | --- | --- | --- |
|  |  |  |  |  |  |  | Lower | Upper |
| Age (years) | .066 | .013 | 24.182 | 1 | .000 | 1.068 | 1.040 | 1.097 |
| ALT (U/L) | .001 | .000 | 4.632 | 1 | .031 | 1.001 | 1.000 | 1.002 |
| ALB (g/L) | -.066 | .027 | 6.162 | 1 | .013 | .936 | .888 | .986 |
| BUN (mmol/L) | .058 | .019 | 8.854 | 1 | .003 | 1.060 | 1.020 | 1.101 |
| Hb (g/L) | -.011 | .006 | 3.100 | 1 | .078 | .990 | .978 | 1.001 |
| SBP (mmHg) | -.016 | .006 | 7.150 | 1 | .007 | .984 | .973 | .996 |
| WBC (109/L) | .066 | .023 | 8.107 | 1 | .004 | 1.069 | 1.021 | 1.119 |
| TG/HDL-C | .151 | .047 | 10.091 | 1 | .001 | 1.163 | 1.059 | 1.276 |

Supplemental table 3 Variate logistic regression of TyG by “Forward stepwise (LR)” method.

|  | B | S.E. | Wald | df | Sig. | Exp(B) | 95% CI for EXP(B) | |
| --- | --- | --- | --- | --- | --- | --- | --- | --- |
|  |  |  |  |  |  |  | Lower | Upper |
| Age (years) | .072 | .013 | 32.499 | 1 | .000 | 1.074 | 1.048 | 1.101 |
| ALT (U/L) | .001 | .001 | 4.946 | 1 | .026 | 1.001 | 1.000 | 1.002 |
| ALB (g/L) | -.090 | .025 | 12.907 | 1 | .000 | .914 | .871 | .960 |
| BUN (mmol/L) | .069 | .018 | 14.463 | 1 | .000 | 1.071 | 1.034 | 1.110 |
| SBP (mmHg) | -.019 | .006 | 10.756 | 1 | .001 | .981 | .970 | .992 |
| WBC (109/L) | .054 | .024 | 5.198 | 1 | .023 | 1.056 | 1.008 | 1.106 |
| TyG | 1.106 | .189 | 34.067 | 1 | .000 | 3.021 | 2.084 | 4.379 |

Supplemental table 4 Variate logistic regression of TyG by “Backward stepwise (LR)” method.

|  | B | S.E. | Wald | df | Sig. | Exp(B) | 95% CI for EXP(B) | |
| --- | --- | --- | --- | --- | --- | --- | --- | --- |
|  |  |  |  |  |  |  | Lower | Upper |
| Age (years) | .069 | .013 | 28.593 | 1 | .000 | 1.071 | 1.044 | 1.098 |
| ALT (U/L) | .001 | .001 | 5.357 | 1 | .021 | 1.001 | 1.000 | 1.002 |
| ALB (g/L) | -.077 | .026 | 8.854 | 1 | .003 | .926 | .880 | .974 |
| BUN (mmol/L) | .061 | .019 | 10.647 | 1 | .001 | 1.062 | 1.024 | 1.102 |
| Hb (g/L) | -.010 | .006 | 2.950 | 1 | .086 | .990 | .978 | 1.001 |
| SBP (mmHg) | -.018 | .006 | 9.908 | 1 | .002 | .982 | .971 | .993 |
| WBC (109/L) | .059 | .024 | 6.153 | 1 | .013 | 1.061 | 1.012 | 1.112 |
| TyG | 1.125 | .190 | 35.073 | 1 | .000 | 3.080 | 2.123 | 4.469 |
